# Optimized artificial neural network application for estimating oil recovery factor of solution gas drive sandstone reservoirs

**DOI:** 10.1016/j.heliyon.2024.e33824

**Published:** 2024-06-28

**Authors:** Muhammad Taufiq Fathaddin, Sonny Irawan, Rini Setiati, Pri Agung Rakhmanto, Suryo Prakoso, Dwi Atty Mardiana

**Affiliations:** a Department of Petroleum Engineering, Universitas Trisakti, 11440 Jakarta, Indonesia; b Department of Petroleum Engineering, Nazarbayev University, 10000 Nur Sultan, Kazakhstan

**Keywords:** Artificial neural network, Recovery factor, Reservoir, Sandstone, Solution gas drive

## Abstract

The most crucial aspect in determining field development plans is the oil recovery factor (RF). However, RF has a complex relationship with the reservoir rock and fluid properties. The application of artificial neural networks is able to produce complex correlations between reservoir parameters that affect the recovery factor. This research provides a new approach to improve the accuracy of the ANN model in the form of steps including removing outlier data, selecting input parameters, selecting transferring functions, selecting the number of neurons, and determining hidden layers. By applying these steps, an ANN model was selected with nine input parameters consisting of oil viscosity, water saturation, initial oil formation volume factor, formation thickness, initial pressure, permeability, specific gravity of oil, porosity, and original oil in place. Furthermore, based on the correlation coefficient, a tangent sigmoid transferring function, 30 neurons, and two hidden layers were determined. The proposed ANN correlation gives the best accuracy compared to the previous correlations. This is proved by the highest correlation coefficient of 0.91657.

## 1 Introduction

The main concern of company management when a new oil reservoir is discovered with an exploratory well is the estimation of the oil that can be obtained by natural mechanisms from the reservoir. The recovery factor (RF) for an oil reservoir is a very important parameter for companies to plan and optimize field development, manage ongoing production, and identify profitable investments among other technical and commercial decisions. This parameter defines oil volume that can be obtained from the initial oil volume in the reservoir [[1], [2], [3], [4]].

In oil and gas projects, determining the recovery factor is one of the biggest uncertainties. Predicting the recovery factor is challenging because many variables affect the oil production from the reservoir. These include variables that are uncertain and beyond the control of oil and gas operators, such as fluid flow in pores, reservoir drive mechanisms, and engineering design-based variables, such as well spacing, well completion, as well as secondary and tertiary recovery mechanisms. In the early life of a field, inadequate production data coupled with subsurface uncertainty make RF predictions uncertain [3]. In general, the factors that affect oil recovery are the physical properties of the reservoir rock and fluid, reservoir drives, and the type of reservoir development [[5], [6], [7]]. Changes in the physical properties of reservoir rocks and fluids to obtain a higher recovery factor can be done using enhanced oil recovery (EOR) methods. EOR is divided into four categories including thermal, chemical, microbial, and miscible methods. These methods are used to remove residual oil that has not moved in the reservoir after the primary and secondary recovery stages [8,9].

Primary recovery factors are known to vary over a wide range. Under the most unfavorable conditions recovery efficiencies as low as 10 percent of the oil in place have been experienced. On the other hand, recovery levels as high as 85 percent to 90 percent of the oil in place have been reported. The main reason for the difference is in the transport mechanism itself – specifically whether oil is displaced by invading water from the aquifer (water drive), by expanding gas from solution (solution gas drive), by expanding gas cap (gas cap drive), or by conducting gravity segregation (segregation drive) [[10], [11], [12]].

Empirical methods can be used at the stage before or after field development. It involves RF estimation through the application of regression equations using parameters that have an impact on oil recovery from multiple reservoirs with similar characteristics. In the early stages of field development, data obtained from direct measurements are not always available. In addition, there may be little or no reservoir performance data available. In this case, the empirical method may provide a quick and easy technique to give an initial estimate of RF with limited data within a given tolerance to provide a framework for investment [13].

In the last decade, various artificial intelligence (AI) techniques have been used to estimate the recovery factor both for water drive reservoirs and solution gas drive reservoirs. AI is able to model complex reservoirs in a relatively short computation time [14,15]. The following is a summary of previous studies.

In 2014, Okpere and Njoku [16] applied artificial neural networks to predict the recovery factor for oil reservoirs in Niger Delta. They divided the data from 94 reservoirs into three groups: 60 % of the data was used for training the ANNs model, 20 % was used for validating the model, and the rest was used for testing the trained model. They used the connate water viscosity, connate water saturation, oil viscosity, oil formation volume factor, initial and abandonment reservoir pressures, permeability, and porosity as input parameters to predict the oil RF. Their model was built for water drive reservoir using backpropagation network.

Noureldien and El-Banbi (2015) proposed two ANN models namely Simple ANN and Sophisticated ANN to predict oil recovery factors for both solution gas drive and water drive reservoirs. The input parameters used to generate the first model were asset area, stock tank original oil in place (STOOIP), net pay thickness, porosity, Lorenz coefficient, initial water saturation, permeability, API, oil viscosity, and reservoir pressure, while additional operational and technological parameters were used for the second model. The first and the second models predicted the RF with an absolute average percentage error (AAPE) of 9.5 % and 8.0 % respectively for the testing dataset [17].

Ahmed et al. (2017) and Mahmoud et al. (2019) applied four artificial intelligence techniques, namely artificial neural networks (ANN), radial basis neuron networks (RNN), adaptive neuro-fuzzy inference systems (ANFIS) with subtractive clustering, and support vector machines (SVM). They used 10 parameters from 130 sandstone water drive reservoirs to determine the oil recovery factor. The ten parameters used in this study were the same as those used by Noureldien and El-Banbi (2015). Furthermore, the extracted weights and biases of the optimized ANNs were used to develop an empirical equation that could be programmed and used for estimating the RF of water drive sandy reservoirs. ANN was the best model of the four artificial intelligence techniques used because this model had the lowest AAPE, namely 7.92 %, and the highest determination coefficient, namely 0.94, for RF prediction on the testing dataset [4,17].

Roustazadeh et al. (2024) used Atlas, GasIS, Commercial, and TORIS databases to construct machine learning models for estimating the recovery factors of oil and gas reservoir. Combinations of the databases and three algorithms i.e., stepwise multiple linear regression (MLR), extreme gradient boosting (XGBoost), and support vector machine (SVM), were applied in the modeling. They found that the XGBoost model predicted both oil and gas RFs more accurately than the SVM and MLR models for the training and testing datasets [18]. In addition, other machine learning methods such as multilayer feedforward neural network (MLFNN), function networks (FNs), and random forest had been used for reservoir characterization [19].

Gomes et al. (2018) used two datasets containing 769 oil-bearing reservoirs in Middle Eastern Fields to discuss the reservoir/field data aggregation process, verification process, and validation process, to extract some key determinant parameters for use in comparison of oil recovery factor (RF) against global analogs. Four data analytics methodologies used in the study were fuzzy logic with backpropagation ANN, symbolic regression with a genetic algorithm, feed-forward backpropagation neural network, and Boruta algorithm with random forest classification method. By using symbolic regression with a genetic algorithm, the recovery factor can be modeled and predicted. The main inputs to this model were 6 independent variables such as oil viscosity, the ratio between oil and water viscosity, the product of average permeability and reservoir thickness divided by oil viscosity, maximum capillary pressure at reservoir top, density of well, and average water saturation. This model gave a training determination coefficient of 0.86 and a testing determination coefficient of 0.62 [20].

Al Tashi et al. (2021) applied ANN for the classification of reservoir recovery factors. They used data obtained from 367 sandstone and carbonate reservoirs with water and solution gas drives. They developed an ANN model with 10 input parameters, namely permeability, oil viscosity at bubble point, connate water saturation, initial reservoir pressure, pressure at the end of primary recovery, oil viscosity at initial pressure, solution gas ratio at abandonment pressure, oil formation volume factor at bubble point pressure, oil formation volume factor at initial pressure, and original oil in place at initial pressure. In the study, the ANN method was equipped with several algorithms such as non-dominated Sorting Genetic Algorithm II, Multi-Objective Gray Wolf Optimizer, and Multi-Objective Particle Swarn Optimization [21].

Makhotin et al. (2022) used tree-based machine learning models to forecast the oil recovery factor for flooding. Two cases from more than 2000 reservoirs were investigated. The first case used parameters related to geometry, storage, geology, transport, and fluid properties, while the second case applied additional parameters including production and development data. The best model had a mean absolute error of 4.91 and a determination coefficient of 0.8 for the testing datasets [22].

The aim of this study was to increase the accuracy of the ANN model in predicting oil recovery factors by selecting the transferring function, number of neurons, and number of hidden layers. Data processing was carried out before modeling, including removing outliers and selecting input parameters. After being validated with data, the proposed correlation was compared with previous regression and ANN methods to compare their accuracy.

## 2 Methodology

The methodology applied for predicting oil recovery factor for solution gas drive reservoirs presented in this study covered four steps, namely data acquisition and screening, parameter selection, development of the ANN model, and statistical evaluation. MATLAB and SPSS software were used as the research tools.

### 2.1 Previous correlations

In 1945 Craze and Buckley collected a large amount of data on the performance of about 103 oil reservoirs in the United States. Twenty-seven of these reservoirs are produced by solution gas drive. The oil recovery factor (RF) equation for reservoir depletion in bbl/acre-ft is as follows [23].

(1) $$\text{RF}=7758\;\varphi \left[\frac{1-S_{w}}{B_{\text{oi}}}-\frac{1-S_{w}-S_{g}}{B_{\text{oa}}}\right]$$

where φ is porosity, S_w_ is water saturation, S_g_ is gas saturation, B_oi_ is oil formation volume factor at initial pressure, and B_oa_ is oil formation volume factor at abandonment pressure.

To obtain the oil recovery factor fraction, the result obtained by calculating Eq. (1) is divided by the initial oil volume. Therefore, the oil recovery (RO) equation is obtained as follows:

(2) $$\text{RO}=\left[1-\frac{\left(1-S_{w}-S_{g}\right)}{\left(1-S_{w}\right)}\frac{B_{\text{oi}}}{B_{\text{oa}}}\right]$$

In 1956, API proposed an empirical equation for oil recovery based on data from eighty solution gas drive reservoirs. Of these, 67 data were from sandstone reservoirs and the rest were from carbonate reservoirs. The published correlations are as follows [2,11]:

(3) $$\text{RO}=3244{\left[\frac{\varphi \left(1-S_{\text{wi}}\right)}{B_{\text{ob}}}\right]}^{1.1611}{\left(\frac{k}{\mu_{\text{ob}}}\right)}^{0.0979}{\left(S_{\text{wi}}\right)}^{0.3722}{\left(\frac{p_{b}}{p_{a}}\right)}^{0.1741}$$

where S_wi_ is initial water saturation, B_ob_ is oil formation volume factor at bubble point pressure, k is permeability, μ_ob_ is oil viscosity at bubble point pressure, p_b_ is bubble point pressure, and p_a_ is abandonment pressure.

In 1967, API proposed another oil recovery equation based on a larger amount of data, namely data from 116 solution gas drive sandstone reservoirs. The published regression equation for the prediction of oil recovery (RO) is [2]:

(4) $$\text{RO}=6533{\left[\frac{\varphi \left(1-S_{w}\right)}{B_{\text{ob}}}\right]}^{1.312}{\left(\frac{k}{\mu_{\text{ob}}}\right)}^{0.0816}{\left(S_{w}\right)}^{0.463}{\left(\frac{p_{b}-p_{a}}{p_{b}}\right)}^{0.1741}$$

Arps (1968) used API data to derive the empirical oil recovery factor equation for a solution gas drive reservoir as follows [11,24]:

(5) $$\text{RF}=41.815{\left[\frac{\varphi \left(1-S_{\text{wi}}\right)}{B_{\text{ob}}}\right]}^{0.611}{\left(\frac{1000k}{\mu_{\text{ob}}}\right)}^{0.0979}{\left(S_{\text{wi}}\right)}^{0.3722}{\left(\frac{p_{b}}{p_{a}}\right)}^{0.1741}$$

or

(6) $$\text{RO}=41.815{\left[\frac{\varphi \left(1-S_{\text{wi}}\right)}{B_{\text{ob}}}\right]}^{0.611}{\left(\frac{1000k}{\mu_{\text{ob}}}\right)}^{0.0979}{\left(S_{\text{wi}}\right)}^{0.3722}{\left(\frac{p_{b}}{p_{a}}\right)}^{0.1741}\left(\frac{\text{OOIP}}{100}\right)$$

Gulstad (1995) proposed an empirical equation of recovery factor using multiple linear regression techniques using the same API data used in previous publications for solution gas drive sandstone reservoirs. The oil recovery factor equation proposed is as follows [2]:

(7) $$\text{RO}=-264.59+0.34\left(\text{OOIP}\right)+29.37\;\ln\left(R_{si}\right)-0.06\left(\frac{k}{\mu_{\text{ob}}}\right)+10.70\;\ln\left(\frac{k}{\mu_{\text{ob}}}\right)-12.64\;\ln\left(h\right)$$

where OOIP is original oil in place, R_si_ is initial solution gas oil ratio, and h is formation thickness.

Onolemhemhen et al. (2016) introduced an empirical equation to estimate the oil recovery factor of dissolved gas drive reservoir. The equation was derived using data from 128 oil reservoirs in the Niger Delta. The equation for the oil recovery factor given is as follows [25]:

(8) $$\text{RF}=C_{f}\left(a\mu_{o}^{-0.1}+b{\text{API}}^{0.487}+cp_{i}^{0.161}-dS_{\text{or}}-1.6{\times 10}^{-8}R_{\text{si}}^{2}+0.2814\right)$$

or

(9) $$\text{RO}=C_{f}\left(a\mu_{o}^{-0.1}+b{\text{API}}^{0.487}+cp_{i}^{0.161}-dS_{\text{or}}-1.6{\times 10}^{-8}R_{\text{si}}^{2}+0.2814\right)\left(\frac{\text{OOIP}}{100}\right)$$

where μ_o_ is oil viscosity, API is specific gravity of oil, p_i_ is initial pressure, S_or_ is residual oil saturation, C_f_ is conversion factor of 1.0001, a = 0.127, b = 0.0218, c = 0.0341, and d = 0.1924.

### 2.2 Data acquisition and screening

**Fig. 1:**
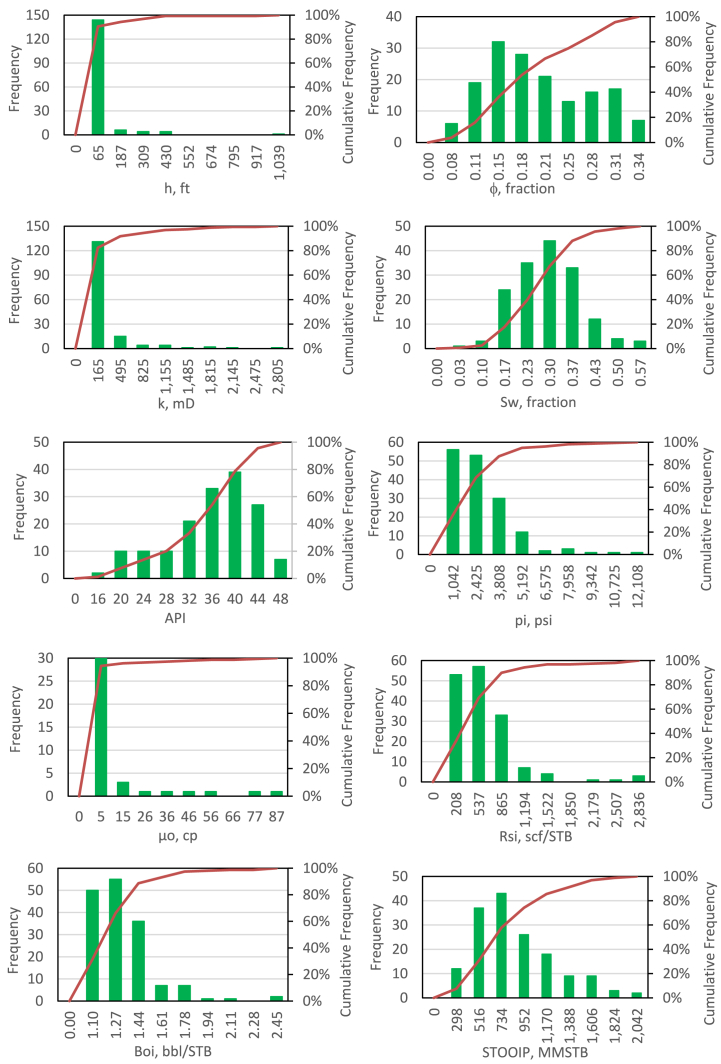
frequency and cumulative frequency for each parameter.

Dataset from 159 solution gas drive reservoirs were collected from Refs. [2,25]. The database were statistically processed. They were grouped into several classes to generate frequency distribution for each parameter. The green histogram graphs in Fig. 1 shows the frequency distributions for all parameters. Based on these frequency distributions, cumulative frequency curves can be determined which ranging from 0 % to 100 %. The cumulative frequency curves are expressed by the red curves in Fig. 1.

In this research, a dataset was designated as outlier if it had at least one parameter that was located in a cumulative frequency interval that was less than 1 % (P1) or located in a cumulative frequency interval that was greater than 99 % (P99). Therefore, P1 and P99 were determined as the lower and upper limits of the database. The values of P1 and P99 for each parameter are given in Table 1.

**Table 1:** determination of upper and lower limits of parameters.

Parameter	P1	P99
**h, ft**	0.73	411.71
**φ, fraction**	0.0217	0.3363
**k, mD**	2.1	1956.4
**S**_**w**_**, fraction**	0.047	0.531
**API Gravity**	12.8	46.8
**p**_**i**_**, psi**	29.6	9010.0
**μ**_**o**_**, cp**	0.06	70.62
**R**_**si**_**, scf/STB**	6.3	2682.5
**B**_**oi**_**, bbl/STB**	0.036	2.312
**OOIP, MMSTB**	40	1823

Removing outlier datasets from the analyzed database aims to obtain a strong relationship between input parameters and output parameters for most of the data. By following this procedure, 27 datasets were determined to be outliers. Therefore, the 159 datasets in the database were reduced to 132 datasets. Although the outlier datasets have been removed, the remaining datasets were believed to still represent all reservoirs.

Table 2, Table 3 present descriptive statistics before and after removing outliers, respectively. Apart from that, the mean, median, and standard error tend to decrease due to the removal process. This indicates that data located in the cumulative frequency interval greater than 99 % (P99) have very large values.

**Table 2:** descriptive statistics before removing outliers.

Parameter	h	φ	k	Sw	API	pi	uo	Rsi	Boi	OOIP	RF
Mean	58.95	0.20	236.48	0.30	35.49	2752	3.68	602.74	1.32	856.30	0.22
Standard Error	9.17	0.01	32.68	0.01	0.60	149	0.90	38.94	0.02	31.31	0.01
Median	25	0.19	83.4	0.3	37	2254	0.95	500	1.262	759.09	0.22
Mode	28	0.15	200	0.3	40	1300	3.00	300	1.26	659.43	0.38
Standard Deviation	115.6	0.071	412.08	0.100	7.557	1883	11.342	491	0.239	394.796	0.10
Sample Variance	13362	0.01	169811	0.01	57.11	3544361	128.64	241116	0.06	155864	0.01
Kurtosis	42.979	−0.786	16.933	0.677	−0.057	7.19	36.26	7.768	6.592	0.552	1.93
Skewness	5.660	0.404	3.716	0.404	−0.676	2.17	5.78	2.365	2.094	0.880	1.09
Range	1095.5	0.295	2969.9	0.6	35.7	12450	91.93	2956	1.51	2141.97	0.56
Minimum	4.5	0.065	0.1	0	14	350	0.07	44	1.02	9.412	0.06
Maximum	1100	0.36	2970	0.6	49.7	12800	92	3000	2.53	2151.38	0.62
Sum	9373.0	32.00	37600	47.35	5642.7	437558	584.55	95835	209.89	136151	35.61
Count	159	159	159	159	159	159	159	159	159	159	159

**Table 3:** descriptive statistics after removing outliers.

Parameter	h	φ	k	Sw	API	pi	uo	Rsi	Boi	OOIP	RF
Mean	45.73	0.19	191.30	0.29	35.73	2592	2.49	548.59	1.30	826.57	0.22
Standard Error	5.67	0.01	25.35	0.01	0.55	124	0.58	30.57	0.02	27.75	0.01
Median	24.8	0.18	77.7	0.3	37	2227	1.00	489	1.261	761.00	0.22
Mode	28	0.15	200	0.3	40	1300	3.00	300	1.26	659.43	0.38
Standard Deviation	65.2	0.066	291.19	0.088	6.295	1424	6.629	351	0.188	318.774	0.10
Sample Variance	4248	0.004	84793	0.01	39.62	2027342	43.94	123391	0.035	101617	0.01
Kurtosis	11.950	−0.733	13.732	−0.449	−0.068	2.42	48.04	5.072	1.687	−0.128	2.28
Skewness	3.361	0.442	3.331	0.181	−0.819	1.27	6.64	1.571	1.220	0.612	1.12
Range	371.5	0.264	1877.8	0.42	26	7750	56.76	2341	0.99	1507.30	0.56
Minimum	4.5	0.065	2.2	0.1	19	600	0.24	44	1.02	189.101	0.06
Maximum	376	0.329	1880	0.52	45	8350	57	2385	2.01	1696.40	0.62
Sum	6035.7	25.48	25251	38.82	4716.2	342205	328.42	72414	172.07	109107	29.42
Count	132	132	132	132	132	132	132	132	132	132	132

**Fig. 2:**
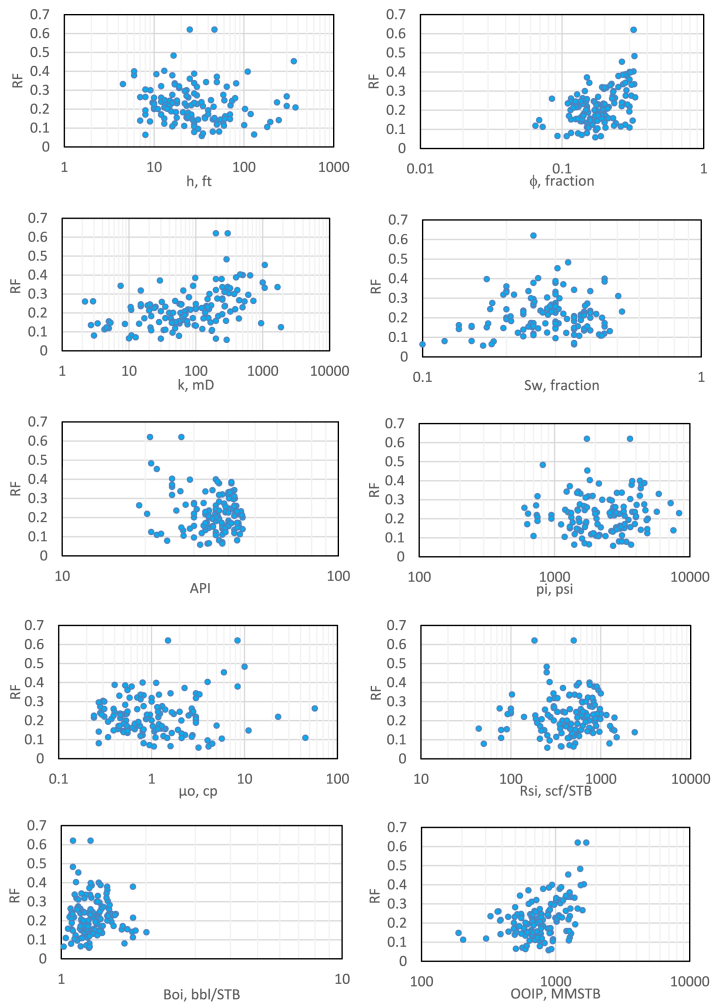
correlation between recovery factor (RF) and each input parameter.

**Fig. 3:**
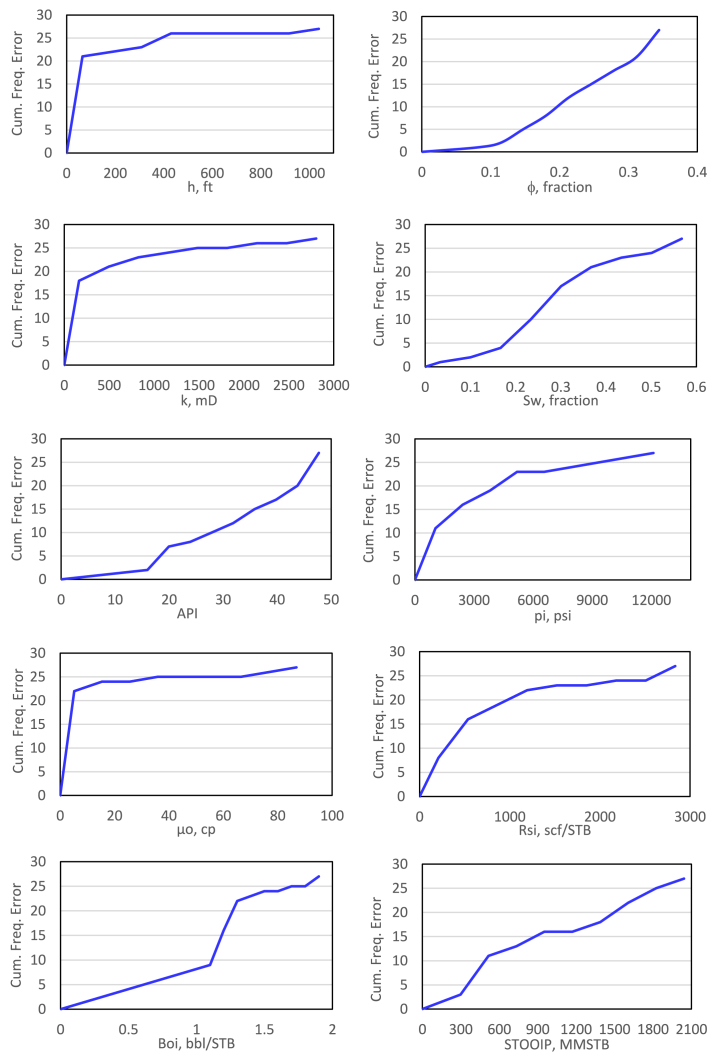
cumulative frequency error for each parameter.

The distribution of data for each parameter regarding the recovery factor after the removal process is explained in Fig. 2. The cumulative frequency error was obtained by comparing the cumulative frequency before and after removing outliers. The cumulative frequency error for each parameter is shown in Fig. 3.

### 2.3 The selection of input parameters

The parameters selected as independent variables should be available at the beginning of the field development since the correlation has been applied to predict the recovery factor as early as possible. In addition, they should have a strong influence on the recovery factor. In this study, the selected parameters were limited by the available data. The parameters used to generate the ANN model as the input parameters or independent variables were formation thickness (h), porosity (φ), permeability (k), water saturation (S_w_), specific gravity of oil (API), initial pressure (p_i_), oil viscosity (μ_o_), solution gas-oil ratio at initial reservoir conditions (R_si_), oil formation volume factor at initial reservoir conditions (B_oi_), and original oil in place (OOIP). Meanwhile recovery factor (RF) was as an output parameter or a dependent variable.

The feedforward backpropagation algorithm was selected for ANN model. The algorithm described the flow of information in an ANN system. The feedforward backpropagation algorithm consisted of two phases, namely forward propagation and backward propagation. The input was fed into the neural network during forward propagation, and the network computes the output. The weights and biases of each neuron were modified during backward propagation to minimize the error between the expected output and the actual output. Levenberg-Marquardt algorithm and gradient descent with momentum weight and bias learning function were chosen as the training function and adaption learning function, respectively.

In the process of building a model with ANN, the input parameters involved need to be normalized, while the output needs to be denormalized. The normalization equation used is as follows [26].

(10) $$x_{j}^{*}=\frac{x_{j}-x_{\min}}{x_{\max}-x_{\min}}$$

where x_j_ and x_j_* are the values measured and normalized at data point j; x_min_ and x_max_ are the minimum and maximum data respectively. The denormalization equation is as follows.

(11) $$x_{j}=x_{j}^{*}\left(x_{\max}-x_{\min}\right)+x_{\min}$$

Various independent variables were combined to be correlated with the dependent variable (RF). The statistical method used to see whether there was a simultaneous influence between the independent variable and the dependent variable was the F test and significance. Based on the F value and significance, the ten best correlations were selected for various numbers of independent variables as shown in Table 4. The next step was to select the best combination of independent variables from the ten combinations of independent variables given in the table. The best combination of independent variables was the combination which given the highest correlation coefficient value. Based on the last column in Table 4, the highest correlation coefficient (R) was resulted from a combination of nine independent variables, namely 0.84074. Therefore, nine independent variables (input parameters) were chosen to form the best ANN model, namely μ_o_, S_w_, B_oi_, h, p_i_, k, API, φ, and OOIP.

**Table 4:** best correlations for various number of independent variables.

No.	Input Parameters	F	Significance	R
1	OOIP	45.27	4.95E-10	0.70918
2	φ, OOIP	27.98	8.04E-11	0.75097
3	log (API), φ, OOIP	18.70	4.02E-10	0.66386
4	k, log (API), φ, OOIP	13.92	1.91E-09	0.75426
5	p_i_, k, log (API), φ, OOIP	11.06	7.66E-09	0.62667
6	log(h), p_i_, k, log (API), φ, OOIP	9.26	2.17E-08	0.71185
7	log (B_oi_), log(h), p_i_, k, log (API), φ, OOIP	8.72	1.11E-08	0.60895
8	S_w_, log (B_oi_), log(h), p_i_, k, log (API), φ, OOIP	8.11	9.44E-09	0.70943
9	μ_o_, S_w_, log (B_oi_), log(h), p_i_, k, log (API), φ, OOIP	7.60	8.49E-09	0.84074
10	log (R_si_), μ_o_, S_w_, log (B_oi_), log(h), p_i_, k, log (API), φ, OOIP	6.80	2.38E-08	0.78385

### 2.4 Optimization of the ANN model

**Fig. 4:**
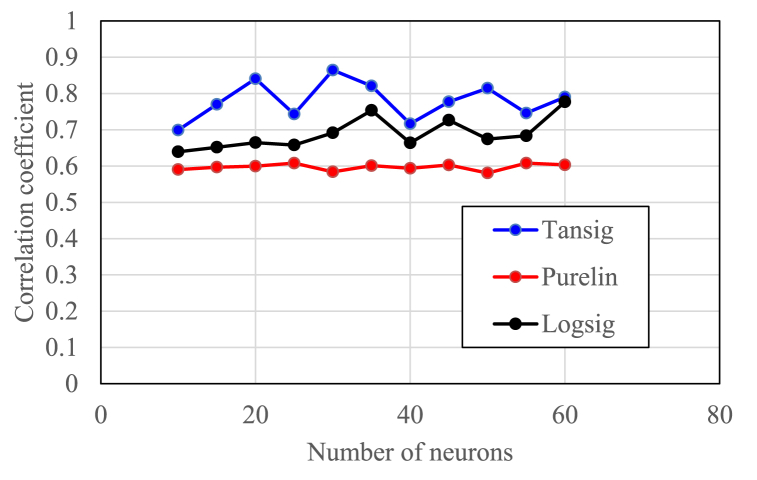
selection of the transferring function and the number of neurons.

Optimization of the ANN model was carried out by selecting the appropriate transfer function, the number of neurons, and the number of hidden layers. The transfer function and the number of neurons per layer were tested based on the correlation coefficient as shown in Fig. 4. There were three transfer functions tested, namely tangent-sigmoid, purelin, and log-sigmoid. The correlation coefficient was used to select the most appropriate transfer function, which provided the best strength of the relationship between input parameters and output parameters.

Based on the curve in the figure, the tangent-sigmoid (tansig) transfer function produced relatively higher correlation coefficient values for a number of neurons of 10–60 compared to other transfer functions. The correlation coefficient values for the tangent-sigmoid transfer function varied between 0.6991 and 0.8645. Therefore, the transfer function was chosen because it produced the most representative model.

Next, the number of neurons that produced the highest correlation coefficient for the tangent-sigmoid function was selected. Based on Fig. 4, the highest correlation coefficient of 0.8645 was obtained with a number of neurons of 30. Therefore, the ANN model was built using 30 neurons.

**Fig. 5:**
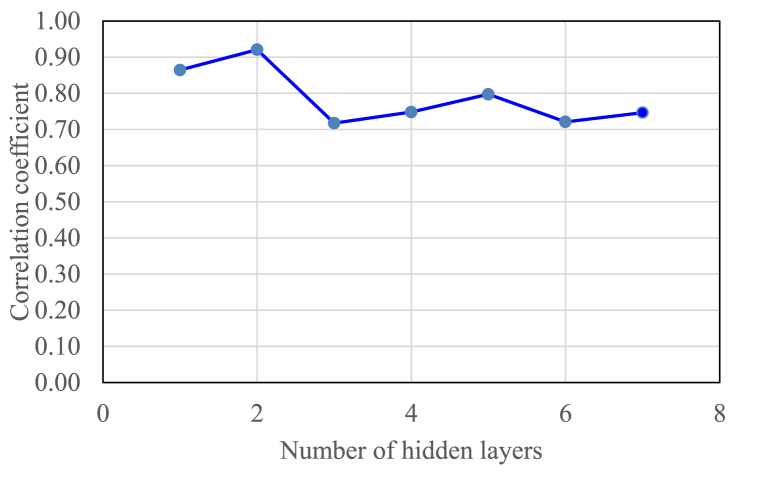
selection of the number of hidden layers using tangent-sigmoid function and 30 neurons.

The next step was to select the hidden layer. The hidden layer selection criterion was also based on the correlation coefficient value. The correlation coefficient value was obtained using Pearson correlation by comparing the output parameters, namely the recovery factor (RF) prediction obtained with the ANN model with the RF obtained from the data [27]. Testing was carried out for the number of hidden layers one to seven. Each test used an ANN model with a tangent-sigmoid transfer function and 30 neurons. Fig. 5 shows the correlation coefficient for various numbers of hidden layers. The image shows that the hidden layers produce correlation coefficients ranging from 0.7175 to 0.9207. The best correlation coefficient was obtained with an ANN model that used 2 hidden layers. Therefore, the optimum ANN model was a model with the tangent-sigmoid transfer function, 30 neurons, and 2 hidden layers.

The use of multiples of five neurons in Fig. 4 aimed to reduce the time to determine the appropriate number of neurons. In addition, assigning the same number of neurons for each hidden layer in this proposed approach was to limit the variation in the number of neurons for each hidden layer. Another problem was that the time complexity increased rapidly with increasing number of hidden layers. Therefore, the number of hidden layers tested in this study was limited to seven. These were the limitations of the application of the proposed steps for finding the optimum ANN model.

## 3 Results and discussion

### 3.1 Correlation validation

**Fig. 6:**
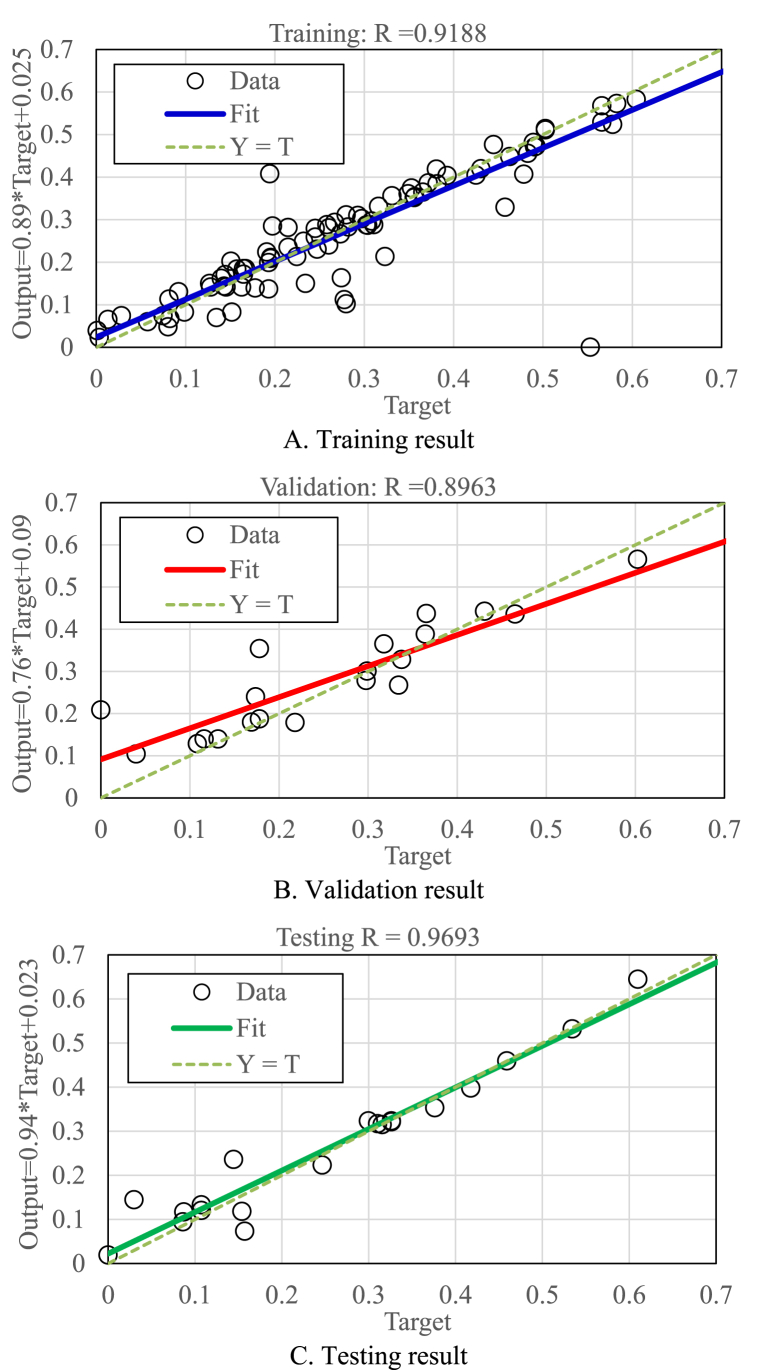
results of the training, validation, and testing of the ANN model.

In order to ensure the feasibility, the proposed correlation had to be validated with actual data. The study used 132 data which were divided into three groups for training, validation, and testing. The percentage of data used for training, validation, and testing was 70 %, 15 %, and 15 %, respectively. Fig. 6 shows the results after the training, validation, and testing processes. The figure indicates that the correlation coefficients for the training, validation, and testing processes are 0.9188, 0.8963, and 0.9693, respectively. Because the correlation coefficient at each stage is close to one, this shows that the ANN model can represent the data very well.

Equation (12) represents the relationship obtained from applying the ANN model. This equation was developed on the same basis as that followed by Mahmoud et al. [4].

(12) $$\text{RF}=\left[\sum_{i=1}^{N}w_{2-i}\text{tansig}\left(\sum_{j=1}^{J}w_{1-i,j}x_{j}^{*}+b_{1i}\right)\right]+b_{2}$$

where RF represents the recovery factor. N and J denote the total neurons in the hidden layer and the total number of input parameters, respectively. w_1_ and w_2_ are the hidden layer weights and the output layer weights, respectively. b_1_ and b_2_ represent the hidden layer biases and the output layer bias, respectively. x* represents the normalized input parameters. The values of the parameters for the model are given in Table 5.

**Table 5:** The proposed ANN-based weights and biases for calculating RF using Equation (12).

	Input layer									Output layer		
No. of Neurons	Weights (w_1_)									Biases (b_1_)	Weights (w_2_)	Bias (b_2_)
	j = 1	j = 2	j = 3	j = 4	j = 5	j = 6	j = 7	j = 8	j = 9			
i = 1	−0.84418	0.64362	0.97660	−0.77133	0.76664	−0.01963	−0.62022	−0.61593	0.48519	2.01730	0.83144	0.08644
i = 2	−0.71873	0.64071	0.13220	0.24668	−1.38000	−0.51766	−0.43257	−0.90720	−0.00748	1.87320	0.54059	
i = 3	0.10614	−0.57218	−0.90265	−0.00821	−0.19444	1.33830	0.45650	−1.00030	0.30282	−1.81460	−0.87687	
i = 4	0.75976	0.70473	−0.38436	−1.17130	−0.75882	−0.16702	0.34875	−0.23246	−0.42903	−1.72470	−0.23915	
i = 5	1.17350	−0.69785	−0.61824	−0.59390	0.86362	−0.97081	−0.90077	−0.25261	−0.38402	−1.19450	0.42640	
i = 6	−0.90927	−0.35426	0.96466	−0.73620	−0.13708	0.66335	0.82958	−0.45975	0.93709	1.42990	−0.00565	
i = 7	1.05260	−0.28971	−0.54674	0.67609	0.91794	−0.22577	0.63922	0.93772	0.13517	−1.22940	−0.60815	
i = 8	1.13250	0.64266	0.98822	−0.61784	−0.95183	−0.81282	−0.08640	−0.15910	−0.09574	−0.85274	0.46263	
i = 9	−0.19842	0.85456	−0.68300	−0.00591	−0.76043	−0.33768	−0.02374	−0.99659	−1.00590	1.04290	0.45917	
i = 10	0.58099	−0.73997	0.65482	−0.83311	−0.76566	−0.91593	−0.01626	0.71836	0.07504	−0.84859	0.15562	
1 = 11	−0.71988	0.15866	−0.40713	0.13659	−1.08640	−0.92967	−0.46538	1.29510	0.30356	0.67264	−0.25104	
i = 12	−0.07931	−0.02210	−0.76424	−0.67526	0.88200	1.56270	−0.11448	0.01113	0.62791	0.49106	−0.07410	
i = 13	1.19580	0.57838	−0.77456	0.59173	0.27046	1.24560	0.06954	−0.83420	−0.06336	−0.55162	−0.50618	
i = 14	1.15480	0.81774	0.39535	0.44067	0.22744	0.17564	0.83750	−0.96912	−0.73163	0.08381	0.29449	
i = 15	0.90375	0.46341	−0.14996	0.37674	−0.64359	−1.01920	0.60842	−0.35531	1.11520	0.03015	−0.37808	
i = 16	0.42063	−0.73429	−0.61959	−0.31393	0.83181	−0.91916	0.41076	0.21284	−1.18940	0.01938	0.76778	
i = 17	−1.21390	0.38541	0.59285	−1.00870	0.72286	−0.40641	−0.07453	−0.47532	0.89350	−0.25237	0.33634	
i = 18	1.07000	0.50092	0.09463	−0.72666	−0.40057	1.05580	0.71922	0.12266	1.33140	0.17721	0.17392	
i = 19	0.92879	−0.77458	0.03658	0.83886	0.02013	−1.27420	0.05989	0.54733	0.57413	0.61551	0.51546	
i = 20	0.34999	−0.90513	0.81074	−0.62524	−0.14804	−1.10560	−0.32354	−0.59298	−0.59539	0.43892	−0.82818	
i = 21	0.52999	0.07803	−0.45654	0.98816	−0.78566	−0.66807	1.19450	−0.81385	−0.34482	0.49533	0.12929	
i = 22	0.52773	1.13420	0.58000	0.08424	−0.64687	0.38604	1.09650	−0.50952	−0.54077	0.98607	−0.26615	
i = 23	−0.33041	−0.48418	0.74464	1.23250	−0.96065	0.58534	0.14433	−0.52522	0.42312	−1.01810	0.10543	
i = 24	0.45582	0.25186	−0.35222	−1.21890	−0.91009	0.42834	0.35012	−0.22297	−1.05150	1.19810	−0.75999	
I = 25	−1.30640	−1.06360	0.41320	−0.26821	−0.98818	−0.06986	0.29085	0.05655	0.60924	−1.20070	0.24431	
I = 26	0.49640	0.61767	−0.94225	−0.84132	−0.19157	0.06662	−0.73951	−0.86280	−0.85725	1.47110	−0.38333	
I = 27	−0.95315	−0.47392	−1.08360	0.73880	−1.19690	−0.36881	−0.41508	−0.43142	0.04254	−1.38070	−0.67536	
I = 28	−0.63229	0.06227	0.28863	−1.38410	1.04600	0.45465	−0.17536	0.97613	−0.18744	−1.59200	0.41785	
I = 29	−1.02280	0.23500	0.64749	0.41865	0.87849	−0.72664	−0.49386	−1.11190	0.44454	−1.80030	−0.21855	
i = 30	−0.84318	0.78292	0.88516	0.65054	−0.01683	0.38908	0.71605	0.88339	0.44212	−2.04140	0.12293	

### 3.2 Comparison to previous correlations

The performance of the proposed correlation was then tested by comparing the use of the correlation to previous correlations in predicting recovery factors. All correlations compared were intended to calculate the recovery factor of solution gas drive reservoirs. Previous correlations involved were API 1956, API 1967, Arps, Gulstad, Onolemhemhen et al., Noureldin and El-Banbi, and Al-Tashi et al. correlations [2,11,17,21,24,25]. The first five correlations used regression method, while the last two correlations used ANN method. The input parameters used by the correlations are given in Table 6.

**Table 6:** comparison of correlation coefficient.

Correlation	Input Parameters	Method	R
API, 1956	φ, μ_ob_, S_wi_, B_ob_, k, p_b_, p_a_	Regression	0.34944
API, 1967	φ, μ_ob_, S_w_, B_ob_, k, p_b_, p_a_	Regression	0.45446
Arps, 1968	φ, μ_ob_, S_wi_, B_ob_, k, p_b_, p_a_, OOIP	Regression	0.52912
Gulstad, 1995	μ_ob_, k, h, R_si_, OOIP	Regression	0.55003
Noureldien & El-Banbi, 2015	A, h, φ, S_wi_, k, API, μ_o_, p, Lc, STOOIP	ANN	0.69155
Onolemhemhen et al., 2016	μ_o_, API, S_or_, R_si_, p_i_, OOIP	Regression	−0.17961
Al-Tashi et al., 2021	k, μ_ob_, S_w_, p_i_, p_ep_, μ_oi_, R_sa_, B_ob_, B_oi_, OOIP	ANN	0.85246
Proposed correlation	μ_o_, S_w_, B_oi_, h, p_i_, k, API, φ, OOIP	ANN	0.91657

**Fig. 7:**
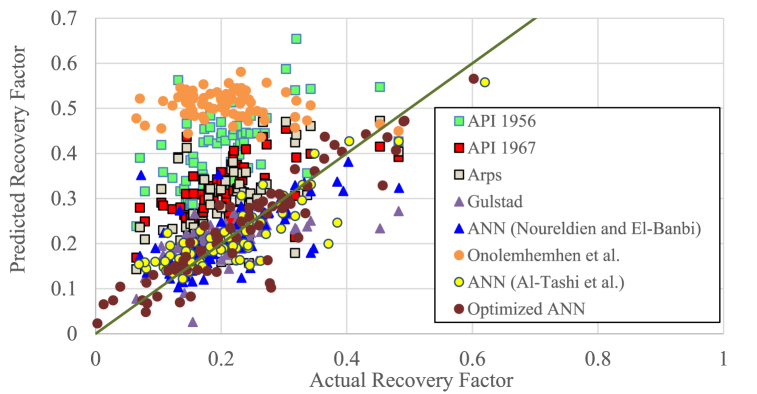
cross-plot of predicted and actual RF using the proposed ANN method and previous methods.

For comparison purpose, 50 % of the 132 data were randomly selected. The prediction results were then compared with actual data to analyze the accuracy of these methods. The comparison is shown in Fig. 7 and Table 6.

Table 6 shows that the method of Onolemhemhen et al. gave a low negative correlation coefficient (R). This shows that there was a very weak correlation between input parameters and RF. Additionally, this method gave predictions that deviated greatly from the data. Onolemhemhen et al. (2016) did not provide any information regarding the type of reservoir formation for their equation [25]. This is believed to drastically affect the accuracy of the model when applied to different environments.

The API 1956 method produced recovery factor predictions that were too optimistic. This is shown by the data plot that lies far above the diagonal line in Fig. 7. Therefore, this method provided a low R as shown in Table 6. Adjustment of the coefficients and constants from the API 1956 equation led to the API 1967 method and Arpps (1968) which generated better recovery factor predictions compared to the previous method. This is indicated by an increase in correlation coefficient. Gulstad (1995) used the parameters of original oil in place, initial solution gas-oil ratio, and formation thickness to replace the parameters of oil formation volume, water saturation, porosity and reservoir pressure, resulting in increased accuracy for predicting RF. The Gulstad method was the best regression method with R of 0.55003.

All the ANN methods involved generated higher accuracy than regression methods. The Noureldin and El-Banbi (2015) method used an ANN model with ten input parameters as described in the introduction. In this study, Lorenz coefficient data were not available, so the Lorenz coefficient was generated randomly with a uniform distribution from 0.25 to 0.6 as specified in reference [17]. As shown in Table 6, this method generated a predicted recovery factor with R of 0.69155.

Al-Tashi et al. (2021) developed an ANN model using 10 input parameters with different combinations of the Noureldin and El-Banbi's correlation (2015) as given in Table 6. In addition, the correlation of Al-Tashi et al. applied the multi objective gray wolf optimizer (MOGWO) algorithm introduced by Mirjalili et al. (2016) [28]. The application of this algorithm produced RF predictions closer to the actual conditions compared to the Noureldin and El-Banbi's method (2015) with a fairly high R, namely 0.85246.

The proposed correlation used an ANN model using 9 input parameters with different combinations from the correlation of Noureldin and El-Banbi (2015) and the correlation of Al-Tashi et al. (2021) as described in Table 6. In addition, this proposed correlation applied the process of selecting the transferring function and optimizing the number of neurons and hidden layers as shown in Fig. 4, Fig. 5. Table 6 indicates that the proposed method had the best performance, since it generated the highest correlation coefficient (0.91657). In addition, almost all of the ANN prediction data lies very close to the diagonal line in Fig. 7 compared to other models. This indicates the importance of carrying out optimization steps as discussed above to improve the accuracy of the ANN model in predicting recovery factors.

### 3.3 Sensitivity analysis of input parameters

**Fig. 8:**
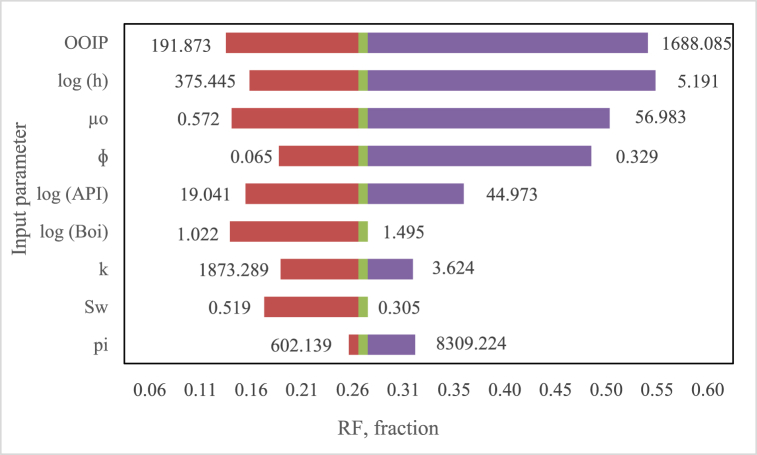
Influence of input parameters on the recovery factor.

There are many parameters that influence oil recovery, from both rock properties and reservoir fluids. The Monte Carlo method was used to analyze the influence of input parameters. All these parameters were varied 300 times randomly using a uniform distribution. The maximum and minimum limits for each parameter were based on the data given in Table 3. After that, ANN was used to predict the output parameters. The relationship between variations in each input parameter and the output parameter is presented in Fig. 8. The figure shows the range of RF values for each parameter. The length of the range shows the magnitude of the influence of the input parameters on RF changes. Based on Fig. 8, it can be seen that the input parameters with the largest to smallest influence on the recovery factor are original oil in place (OOIP), formation thickness (h), oil viscosity (μ_o_), porosity (φ), specific gravity of oil (API), oil formation volume factor at initial reservoir conditions (B_oi_), permeability (k), water saturation (S_w_), and initial pressure (p_i_), respectively.

The level of influence of input parameters (independent variables) on the recovery factor (dependent variable) is unique. It depends on depositional environment, drive mechanism, rock and fluid properties, and driving mechanisms [13,29]. Previous studies showed different levels of influence of input parameters on the recovery factor as shown in Table 7. Okpere and Ndibueze (2013) analyzed the influence of input parameters on recovery factors for 40 strong water-driven reservoirs with sandstone lithology in the Niger Delta. The results of their study showed that the pressure drop (p_i_/p_a_) was the most sensitive input parameter that affected the recovery factor. The analysis results are shown in the third column of Table 7 [13]. Babayeva (2019) analyzed parameters that were sensitive to recovery factors in the Guneshli offshore field. The field was divided into 10 isolated blocks. In addition, the Guneshli field consisted of eight layers. Sensitivity analysis indicated that several layers have different sequences of parameters that influence the recovery factor. The complete results are shown in the fourth to sixth columns in Table 7 [30].

**Table 7:** influence level of input parameters on recovery factor from largest to smallest.

Influence Level	This Study	Okpere & Ndibueze 2013 [13]	Babayeva, 2019 [30]		
			Layer A	Layer C	Layer E
1	OOIP	p_i_	A	h	h
2	h	p_a_	h	A	S_o_
3	μo	S_w_	ρ_o_	φ	A
4	φ	μ_oi_	φ	S_o_	φ
5	API	μ_wi_	S_o_	ρ_o_	ρ_o_
6	B_oi_	k			
7	k	φ			
8	S_w_	B_oi_			
9	p_i_				

## 4 Conclusions

Based on the results and analysis discussed above, several conclusions can be drawn as follows. An artificial neural network (ANN) can be applied to predict recovery factors for solution gas drive sandstone reservoirs. Correlation accuracy can be determined based on a comparison of the statistical parameters correlation coefficient (R). The Gulstad method is the best regression model with R of 0.55003. All correlations using the ANN model show better accuracy compared to correlations using regression model. Removing outliers, selecting input parameters, selecting the number of neurons, selecting transfer function, and selecting number of hidden layers are necessary to optimize the accuracy of the ANN model. The optimized ANN model provides better accuracy compared to previous ANN models. The proposed correlation has the highest R of 0.91657. In addition, according to the sensitivity analysis, the initial oil in place parameter had the greatest influence on the recovery factor. The limitation of applying the approach proposed in this research which will become a challenge in the future is the calculation time. Calculation time problems are caused by variations in the number of neurons and model complexity due to the number of hidden layers.

## Data availability statement

Data can be obtained from Refs. [2,25].

## CRediT authorship contribution statement

**Muhammad Taufiq Fathaddin:** Writing – original draft, Supervision, Methodology, Funding acquisition, Formal analysis. **Sonny Irawan:** Writing – review & editing, Supervision, Resources. **Rini Setiati:** Writing – original draft, Project administration, Formal analysis, Conceptualization. **Pri Agung Rakhmanto:** Visualization, Validation, Software, Data curation. **Suryo Prakoso:** Writing – review & editing, Visualization, Data curation. **Dwi Atty Mardiana:** Writing – original draft, Validation, Investigation.

## Declaration of competing interest

The authors declare that they have no known competing financial interests or personal relationships that could have appeared to influence the work reported in this paper.

## Nomenclature

**Symbols**
A: reservoir area [acre]
API: specific gravity of oil [°]
B_o_: oil formation volume factor [bbl/STB]
B_oa_: B_o_ at abandonment pressure [bbl/STB]
B_ob_: B_o_ at bubble point pressure [bbl/STB]
B_oi_: B_o_ at initial pressure [bbl/STB]
b_1_: hidden layer biases [dimensionless]
b_2_: output layer bias [dimensionless]
h: formation thickness [ft]
J: total number of input parameters [dimensionless]
k: permeability [mD]
Lc: Lorenz coefficient [fraction]
N: total neurons in hidden layer [dimensionless]
OOIP: original oil in place [STB]
p: reservoir pressure [psig]
p_a_: abandonment pressure [psig]
p_b_: bubble point pressure [psig]
p_ep_: pressure at end of primary recovery [psig]
p_i_: initial pressure [psig]
RF: recovery factor [dimensionless]
R_sa_: solution gas oil ratio at p_a_ [SCF/STB]
R_si_: initial solution gas oil ratio [SCF/STB]
S_g_: gas saturation [dimensionless]
S_or_: residual oil saturation [dimensionless]
STOOIP: stock tank original oil in place [STB]
S_w_: water saturation [dimensionless]
S_wi_: initial water saturation [dimensionless]
w_1_: hidden layer weights [dimensionless]
w_2_: output layer weights [dimensionless]
x*: normalized input parameter

**Greek letters**
φ: porosity [dimensionless]
μ_o_: oil viscosity [cp]
μ_ob_: μ_o_ at bubble point pressure [cp]
μ_oi_: μ_o_ at initial pressure [cp]
